# Aqueous copper bioavailability linked to shipwreck-contaminated reef sediments

**DOI:** 10.1038/s41598-019-45911-8

**Published:** 2019-07-02

**Authors:** Adam Hartland, Rebecca Zitoun, Rob Middag, Sylvia Sander, Alix Laferriere, Huma Saeed, Sharon De Luca, Philip M. Ross

**Affiliations:** 1 Environmental Research Institute, School of Science, University of Waikato, Private Bag 3105, Hamilton, 3240 New Zealand; 2 Department of Chemistry and Centre for Trace Element Analysis, University of Otago, PO Box 56, Union Place, Dunedin, New Zealand; 3 Department of Ocean Systems (OCS), NIOZ Royal Netherlands Institute for Sea Research, and Utrecht University, P.O. Box 59, 1790 AB Den Burg, Texel, The Netherlands; 4 Marine Environmental Studies Laboratory, IAEA Environment Laboratories, Department of Nuclear Sciences and Applications, International Atomic Energy Agency, 98000 Monaco, Monaco; 5 Boffa Miskell Ltd, 116 Cameron Rd, Tauranga, 3141 New Zealand

**Keywords:** Ecology, Community ecology, Marine chemistry, Pollution remediation

## Abstract

Pollution from the grounding or sinking of ships can have long lasting effects on the recovery and dynamics of coastal ecosystems. Research on the impact of copper (Cu) pollution from the 2011 *MV Rena* shipwreck at the Astrolabe Reef (Otaiti), New Zealand, 5 years after the grounding, followed a multi-method and multi-disciplinary approach. Three independent measures of aqueous Cu using trace-element-clean-techniques substantiate the presence of high total, total dissolved (<2 µm) and elevated bioavailable Cu in the water column immediately above the aft section of the wreck where the highest sedimentary load of Cu was located. Intermittently elevated concentrations of strong Cu-binding ligands occurred in this location, and their binding strength was consistent with ligands actively produced by organisms in response to Cu induced stress. The recruitment of benthic invertebrates was modified at the high-Cu location. Taxonomic groups usually considered robust to pollution were restricted to this site (e.g. barnacles) or were the most abundant taxa present (e.g. foraminifera). Our results demonstrate that Cu-contaminated sediments can impose a persistent point source of Cu pollution in high-energy reef environments, with the potential to modify the composition and recovery of biological communities.

## Introduction

Between 2004 and 2014, 1,271 ships were lost at sea^1^. The causes may have included collision, adverse weather, loss of stability due to cargo movement or rupture of cargo tanks, explosions, uncontrollable fire, piracy, inadequate vessel maintenance and crew negligence or incompetence. While the environmental effects and legacies of most lost ships are unknown, contamination and subsequently environmental degradation are certain to occur. Metals and other potential contaminants, both organic and inorganic, are present in almost every part of a modern ship, including antifouling paints, electrical and electronic equipment, the hull and other structural components and sacrificial anodes together with fuels, lubricating and hydraulic fluids^2^. For cargo ships, the range of potential contaminants is greater still because these vessels can transport a variety of commercial and industrial chemicals and materials, raw minerals, oils, paints, plastics, manufactured goods, agricultural and horticultural produce, and personal belongings.

Vessels are typically lost in deep water where the costs and practicalities of salvage or monitoring are prohibitive, or they ground on coastlines of nations where environmental regulations are such that monitoring is limited or non-existent^3^. Occasionally, shipwrecks do occur on coastlines where there are public or governmental expectations around gaining an understanding of the environmental consequences of maritime casualties. However, unless a wreck occurs in exceptionally benign or accessible waters, it can be difficult to apply the methods that would normally be used to assess pollution and environmental impacts. As such, the ecological consequences of most shipwrecks are unknown.

**Figure 1:**
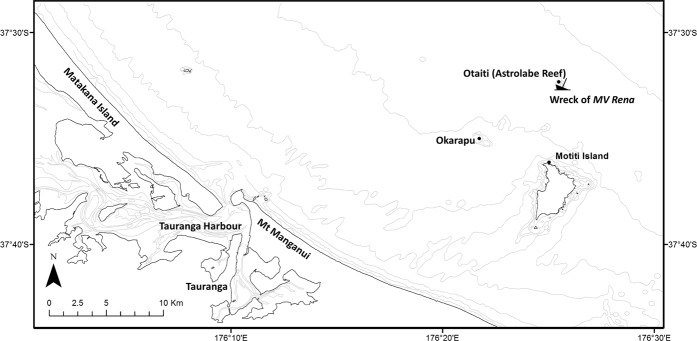
Map of the Bay of Plenty showing the location of the *MV Rena* on Otaiti (Astrolabe Reef),Okarapu (Control site), Motiti Island and Tauranga.

**Figure 2:**
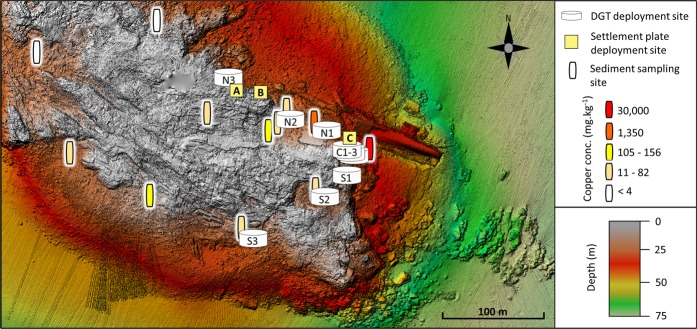
Chart of Otaiti showing the locations of water sampling and DGT deployments (discs) and settlement plate deployments (squares) along the 25 m depth contour. The Cu concentration measured in reef sediments is shown by the coloured cylinders (data from Ross, *et al*.^7^). Elongate remnants of the wreck lie on the central reef (south of settlement plate A), overlap with the northeast corner of the reef and lie parallel to its northeastern edge.

One example of a relatively accessible shipwreck is the *MV Rena*^4^, which grounded and sank in 2011 at Otaiti (Astrolabe Reef; Fig. 1), in New Zealand’s Bay of Plenty. The sinking of the *MV Rena* generated headlines around the world and has been widely reported as New Zealand’s ‘worst maritime environmental disaster’^5^. Potentially because of the rarity of large shipwrecks in New Zealand’s recent maritime history, or because of New Zealand’s “clean green” image, the impacts of the *MV Rena* on Otaiti, both physical and chemical (the latter determined through analysis of sediments and biota^6–8^), have been remarkably well documented^8^. Otaiti lies 25 km offshore from Tauranga, one of New Zealand’s busiest ports. The reef is a high-energy environment, with a mean annual significant wave height of 1.19 m, with significant wave heights of 5.5 m occurring at a ~6-year Annual Return Interval^9^. Otaiti is a pinnacle of rock, which rises from a depth of around 70 m with a small section of reef (15 to 25 m^2^) breaking the water’s surface between mid and low tide. The reef has a base circumference of about 1 km and covers an estimated 461,587 m^2^ of seafloor. The major components of the shipwreck lie at depths ranging from −3 m to −60 m (Fig. 2). These physical and environmental factors have made it challenging to conduct both salvage and environmental monitoring activities. An extended salvage and recovery effort did much to remove contaminated reef sediments and wreckage^8^, yet a legacy of sediment contamination remains. Dempsey, *et al*.^6^ demonstrated both the contaminant legacy of the *Rena* and the usefulness of Diffuse Gradients in Thin Film (DGT) passive samplers for identifying and locating contaminants. Perhaps of greatest interest in trying to understand the long-term chemical and ecological impacts of the *Rena* is the fate of some 7 to 12 tonnes of granulated Cu (clove grade)^2,4–8,10^ which remain trapped beneath the wreck.

Aquatic pollutants, including Cu, have the potential to adversely influence the recovery of marine ecosystems through sub-lethal or lethal effects on resident communities^11–13^ or by altering the rates or locations at which recruitment of early life stages occurs^14^. This latter process can happen through several mechanisms. One is that the free-swimming larvae of benthic species may use water or substrate chemistry to select a settlement location, thereby selecting against contaminated substrate^14^. Alternatively, recruitment may occur despite differences in substrate chemistry (or that of the surrounding water), but subsequent survival rates diminish due to environmental or chemical differences, or through chemistry-dependent changes in (biological) species interactions^14,15^.

In order for Cu to be harmful and toxic, it needs to be present in a bioavailable form to enter the body of an exposed organism and interact with the surface or interior of their cells^16^. According to the literature^17,18^ the bioavailability of Cu is primarily related to the labile inorganic Cu fraction (Cu’), consisting of free Cu ions (Cu^2+^) and Cu complexed to inorganic ligands (CuX_IN_), rather than the total ([Cu_T_]) or total dissolved Cu concentration ([_d_Cu_T_]) in a system^19–21^. Some forms of organically complexed Cu are, however, also considered bioavailable^22^, but it is generally accepted that Cu’ represents the most readily bioavailable and thus toxic form of Cu to marine organisms. Consequently, the labile inorganic Cu fraction (Cu’) is a reasonable indicator of Cu bioavailability and is thus hereinafter referred to as bioavailable Cu (Cu’). The aquatic lifetime of bioavailable Cu (Cu’) (sum of Cu^2+^ and CuX_IN_) is considered low due to organic Cu-complexation (CuL) processes (>99%)^11^, but minor amounts of free Cu^2+^ ions and labile inorganic and organic Cu complexes can still adversely affect exposed organisms. Furthermore, the uptake of Cu’ by organisms will cause the dissociation of CuL due to the perturbation of the equilibrium between CuL and its products (Cu^2+^ +L^−^). Additionally, ligands can be oxidised microbiologically or photo-chemically^23^, thereby changing the relationship between Cu concentration, speciation (i.e. chemical form) and toxicity.

**Figure 3:**
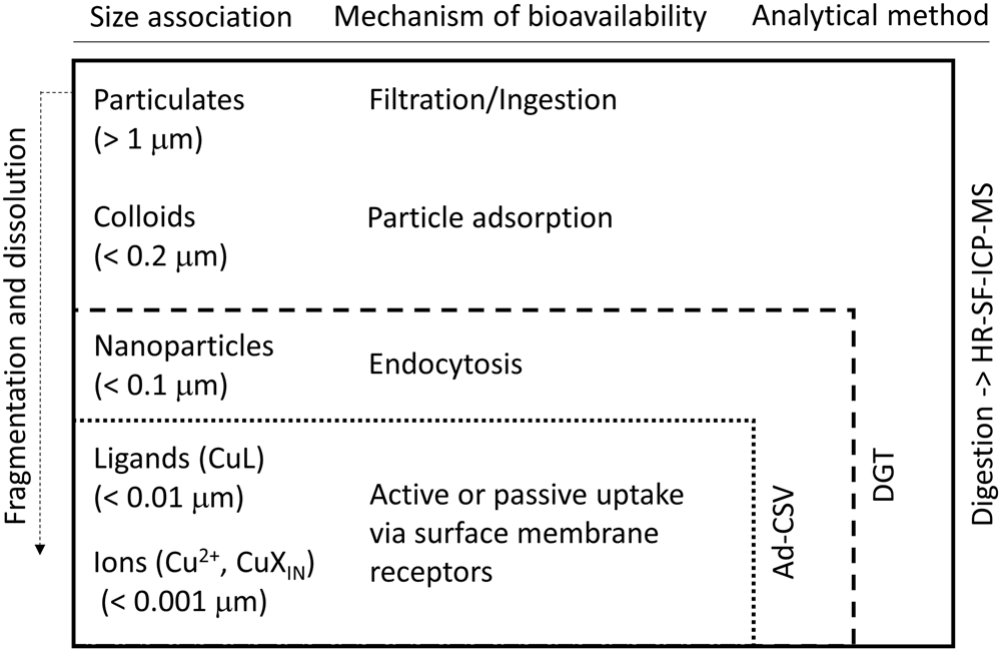
Conceptual figure of the three complementary analytical techniques used to analyse the concentration, speciation, and bioavailability of Cu in the study area of the *MV Rena* at Otaiti (Astrolabe Reef). Depicted AdCSV measurements reflect the Cu speciation analysis, not the _d_Cu_T_ evaluation.

Given the complex interplay between Cu speciation, environmental conditions and toxic effects, it was deemed necessary to expand on the work previously conducted at Otaiti^6^ to examine the aquatic speciation of Cu in the water column and the spatial scale at which water chemistry was affected (site C, Fig. 2)^6^. This study set out to assess the extent of water column Cu contamination at Otaiti, ascertain its bioavailability, and explore, using settlement plates, the effects of Cu contamination on the recruitment of benthic invertebrates^14,24,25^. A multi-method approach was implemented in order to obtain an integrated picture and provide complementary data on the concentration, speciation, distribution, as well as bioavailability of Cu in the working area (Fig. 3), thereby increasing the robustness of the results and conclusion. The multi-method analytical approach consisted of trace element clean sampling and analysis protocols of the Otaiti water column to collect Cu samples for High Resolution Sector Field Inductively Coupled Plasma Mass Spectrometery (HR-SF-ICP-MS) analysis, adsorptive cathodic stripping voltammetry (AdCSV)^26^ with salicylaldoxime (SA) as the complexing agent, and *in-situ* immersive measurements by diffusive gradients thin films (DGT) (see Section 6.1). HR-SF-ICP-MS was used to quantify total Cu (non-filtered, Cu_T_) concentrations, while AdCSV was implemented to measure total dissolved Cu (filtered at 0.2 µm, _d_Cu_T_) concentrations and Cu speciation parameters (e.g. CuX_IN_, Cu^2+^, L, and log*K*) of the collected water samples. DGTs were deployed to obtain a temporal integrated view of bioavailable Cu (Cu_DGT_) in the system (see Section 6.1). We define the following terms for different components of Cu in our analysis: [] denote concentration, inorganically bound Cu (CuX_IN_), hydrated ‘free’ Cu ions (Cu^2+^), Cu-binding organic ligands (L), conditional stability constant of CuL complexes (log*K*), total Cu (non-filtered, Cu_T_), total dissolved Cu filtered at 0.2 μm (_d_Cu_T_), bioavailable Cu (Cu’), and DGT-labile Cu (Cu’+ dissociating Cu^2+^ from complexes with L) reported as Cu_DGT_ calculated using standard DGT theory^6^.

## Results

### Cu concentrations and DGT measurements

The results of the aqueous [Cu] measurements are given in Table 1.

**Table 1:** Averaged total (n = 2), total dissolved (n = 2) and DGT-labile (n = 4) Cu concentrations in Otaiti and Okarapu Reef (control site) water samples collected over two sampling campaigns on 15–16^th^ and 22–23^rd^ June, 2016.

Site	Site label used in Fig. 2	Cu_T_ [nM] HR-SF-ICP-MS	1σ	_d_Cu_T_ [nM] AdCSV	1σ	Cu_DGT_ [nM]	1σ
Control 1	—	1.3	0.3	6.6	1.3	5.4	5.0
Control 2	—	2.7	1.2	5.7	0.8	4.8	0.6
Control 3	—	1.1	0.3	5.4*	0.6	4.9	3.1
North 1	N1	21.0	4.6	5.4*	3.9	3.0	1.7
North 2	N2	1.7	0.5	5.1	2.4	2.2	1.1
North 3	N3	53.9	70.0	8.3	0.1	2.4	0.8
Centre 1	C1	528.0	731.0	10.6	0.3	37.6	38.2
Centre 2	C2	1239.9	869.3	14.3	5.1	33.2	16.7
Centre 3	C3	240.0	97.7	9.2	0.4	17.4	18.6
South 1	S1	9.1	6.9	7.4	4.5	4.6	2.4
South 2	S2	2.8	1.0	6.6	2.1	2.5	0.2
South 3	S3	4.2	0.1	6.7	3.3	1.4	0.2

Note that with the exception of the DGT results the data are the averages with associated standard deviations (σ) of individual samples collected from the same locations on the two separate dates; whereas the DGT error is the standard deviation of four solution probes deployed in tandem. Total Cu (unfiltered) was determined by HR-SF-ICP-MS and total dissolved Cu (at 0.2 µm filtered) by AdCSV. Results marked (*) were from individual sample analyses. Note that the _d_Cu_T_ and _d_Cu_DGT_ concentrations determined by AdCSV and DGT, respectively, are sometimes higher than the Cu_T_ concentrations determined by HR-SF-ICP-MS, which could be a result of sample contamination during sampling and handling, an issue of differing resin capacities of the 4 replicate DGTs, could indicate sediment deposition on some DGTs, or (more likely) is due to spatiotemporal variability in Cu across a dynamic reef environment during the sampling process.

**Figure 4:**
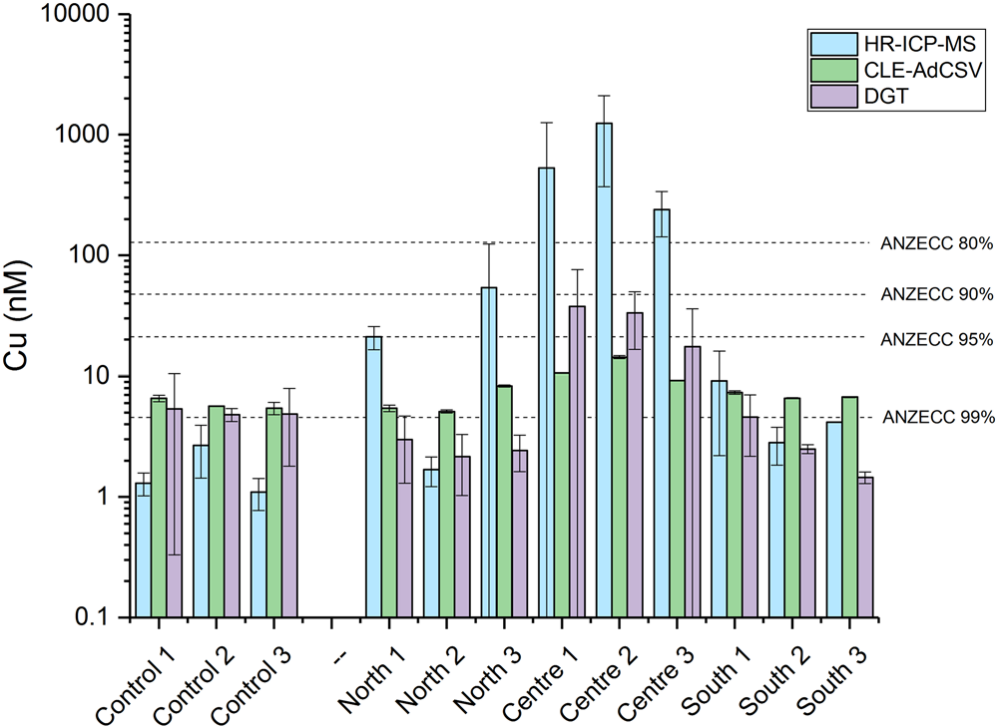
Averaged [Cu_T_] determined by HR-SF-ICP-MS, [_d_Cu_T_] determined by AdCSV in water samples collected in June 2016, and [Cu_DGT_] determined from multi-day DGT deployments at Otaiti and Okarapu Reef (control site) in June 2016. Error bars represent the ±1 σ values for measurements carried out on two separate days (i.e. the inter-sample variability rather than analytical errors). [Cu_DGT_] values are calculated from the average of four DGT Chelex-100 probes deployed between three and four days (*n* = 4). Dashed lines show the Australian and New Zealand Environment and Conservation Council^75^ water quality guidelines for total dissolved Cu concentrations ([_d_Cu_T_]) to protect 99–80% of species.

The [Cu_T_], [_d_Cu_T_] and [Cu_DGT_] fractions varied coherently across the sample set (Table 1; Fig. 4) with markedly higher Cu concentrations encountered at the centre site. The results of these measurements were generally consistent with the known hierarchy of detection between techniques^27^ and indicate that coarse colloids and particles with diameters >0.2 μm were involved in Cu adsorption and mobilisation, driving the exceptionally large variability seen in [Cu_T_] between the sampling campaigns at sites C1-3 (Table 1). We suggest that the dynamic variability in the concentration of colloidal/particulate Cu in these coastal settings also led to disparities between samples collected contemporaneously for Cu_T_ and _d_Cu_T_. These disparities were evident at sites away from the major contamination plume where concentrations of suspended Cu-particles were at their highest (C1-3; Fig. 2, Table 1).

Despite predictable differences between techniques, the overall trend between sites was captured by all three methods. In particular, the Centre sites, located in the vicinity of the Cu deposit, were the most contaminated, consistent with expectations based on previous site assessments^7^ which documented extremely high Cu sediment loads (up to 780 g kg^−1^) in this location (Fig. 2). Clearly, entrainment of this sediment by wave action creates the release of dissolved Cu and the resuspension of Cu-bearing particles in the vicinity of the *Rena* debris (sites C1-3; Table 1).

The overall distributions of [Cu_T_], [_d_Cu_T_] and [Cu_DGT_] reflected a pronounced, but localised contamination at the centre sites (C1-3; Figs 2,3), when compared to the Cu concentrations at the South, North and Control sites. This analysis demonstrates significant Cu enrichment in the water column at Otaiti, with a localised contamination in a relatively small area in the immediate vicinity of the Cu-contaminated sediments (Fig. 3).

DGT is a dynamic method which measures the Cu fraction capable of diffusing within the DGT hydrogel across a 0.45 µm polycarbonate filter membrane^28,29^, i.e. consisting of small colloids, nanoparticles and labile Cu-ligand complexes. Only Cu fractions, which dissociate and subsequently bind with the Chelex-100 chelating resin inside the DGT housing within the timeframe of the deployment contribute to the final measurement of [Cu_DGT_]. This means that DGT measurements may be the most analogous to the steady-state bioavailable Cu^30^ in a given aquatic system, except where Cu is made bioavailable by direct ingestion of Cu particles (e.g. bivalves). The dynamic and time-integrated nature of DGT measurements contribute to the higher values of [Cu_DGT_] compared to [_d_Cu_T_]. DGT also has the advantage of avoiding artefacts arising from sampling and sample handling^27^, although care should still be taken at all stages of associated laboratory operations^31^. Because of the differences in dynamic features between techniques and the potential for analytical artefacts to arise, the multi-method approach to determining aquatic trace metal speciation^27^ adopted here allows us to characterise the system more fully.

The difference between [Cu_T_] and [Cu_DGT_] highlights the fact that a large fraction of [Cu_T_] was accounted for by Cu-bearing particles with diameters >0.1 μm. The residual between [_d_Cu_T_] and [Cu_DGT_] is therefore likely to reflect the presence of partially-labile organic Cu complexes which contributed to the time-integrated DGT measurements. The results of the AdCSV Cu speciation measurements are given below and give appropriate context to this finding.

### Cu speciation by AdCSV

Samples analysed by AdCSV showed the best fit to a one-ligand model and all titrations demonstrated the presence of one organic ligand class with remarkably uniform Cu-binding capacities of log*K* 11.6 ± 0.3 throughout the study area for both sampling periods, despite the highly dynamic nature of the reef environment.

**Figure 5:**
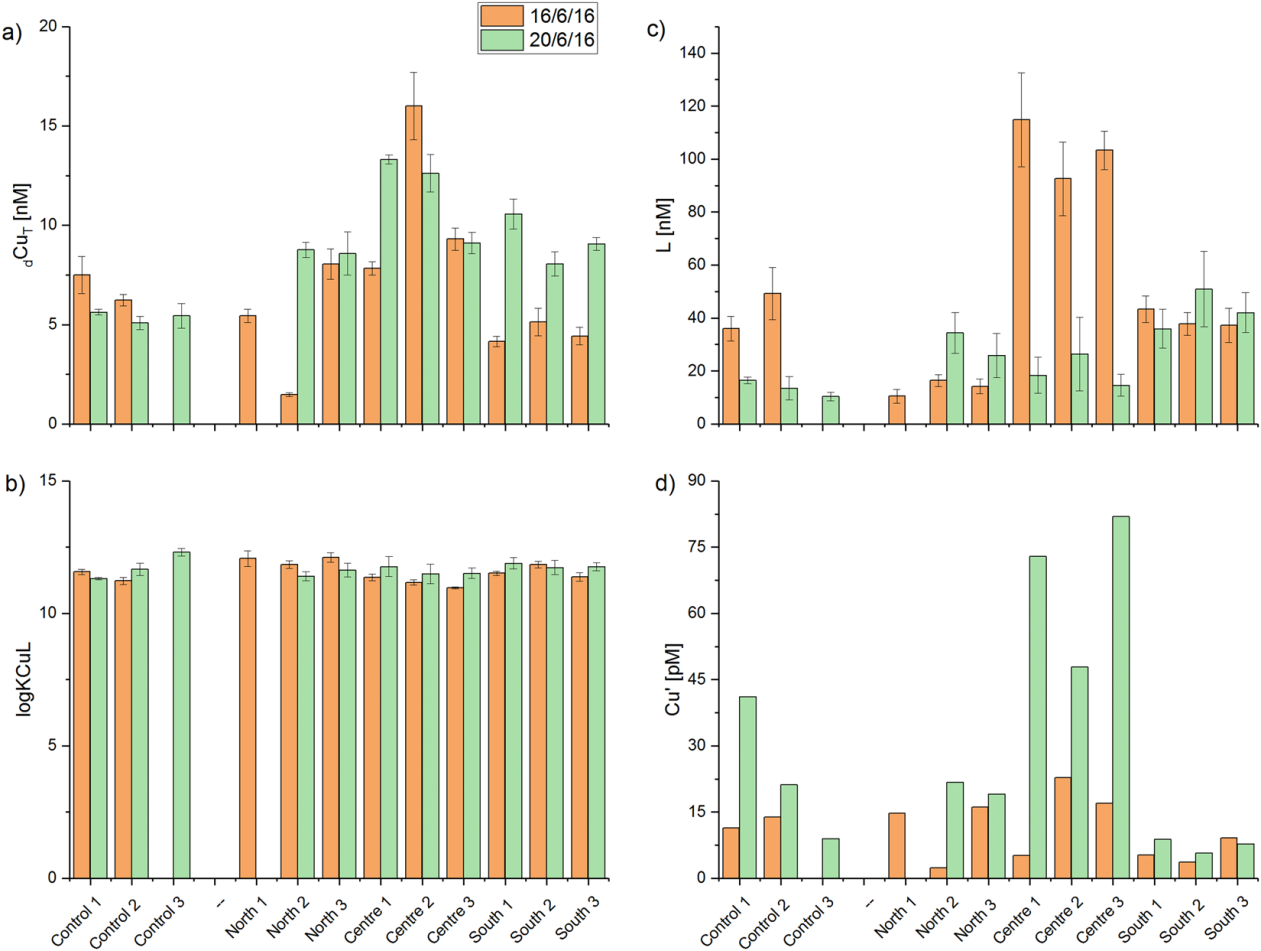
Results of AdCSV measurements detailing total dissolved Cu concentrations ([_d_Cu_T_]) and the properties of natural organic Cu-binding ligands in samples from Otaiti and Okarapu Reef (control site) collected on 16^th^ June 2016 (orange columns) and 20^th^ June 2016 (green columns) including ligand concentrations ([L]), conditional stability constants of ligand-Cu (CuL) complexes (log*K*), and the calculated bioavailable Cu concentration ([Cu’]). Error bars are ±1σ.

**Figure 6:**
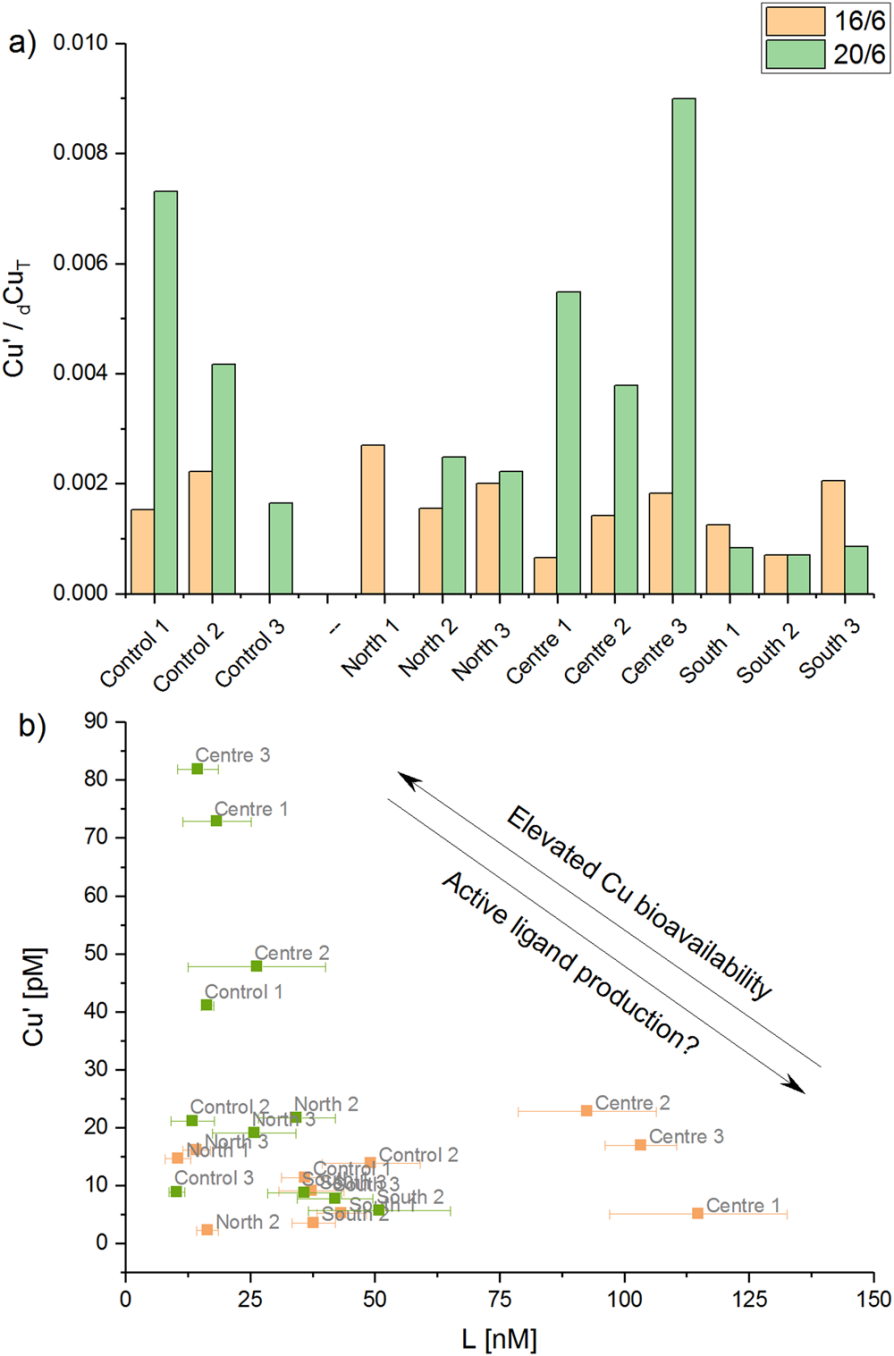
Exploration of AdCSV data: (**a**) Ratio of [Cu’] to [Cu_T_] in water samples collected from Otaiti and Okarapu Reef (control) on the 16^th^ June 2016 (orange columns) and the 20^th^ June 2016 (green columns); and (**b**) relationship between Cu-binding ligand concentration and [Cu’].

The average conditional stability constant (log*K*) on the 16^th^ of June was 11.5 ± 0.4 and 11.7 ± 0.3 on the 20^th^ of June. Ligand concentrations ranged from as low as [L] = 10.3 ± 1.5 nM for the control station (Okarapu Reef) to 114.8 ± 17.8 nM for the stations adjacent of the *MV Rena* wreck (sites C1-3). The ligand concentrations were always in excess of the total dissolved Cu-concentrations (Fig. 5) but were markedly higher on the first sampling trip (16^th^ June) at sites C1-3, relative to the second trip (20^th^ June). As would be expected, the availability of strong Cu binding ligands in the reef debris field (sites C1-3; Fig. 2) was a direct determinant of the bioavailability of Cu in this location (Fig. 6b). At low ligand concentrations, Cu was more bioavailable at the centre sites and vice versa (Fig. 6b). Bioavailable Cu-concentrations in solution were low (relative to total), ranging from 2.3 to 81.9 pM or <1% of [_d_Cu_T_] (Figs 5,6a), being an order-of-magnitude lower than DGT measurements (Fig. 4). As expected from sediment measurements, the highest [Cu’] were detected at sites C1-3 adjacent to the *Rena* wreck, with average concentrations of 15 ± 9 pM on the 16^th^ of June and 67.5 ± 17.7 pM on the 20^th^ of June. It is important to note that water samples for AdCSV analyses were collected under quiescent ocean conditions, whereas DGT probes measured Cu fluxes under all conditions over the deployment period. Therefore, the DGT probes were likely to have accumulated Cu under more turbulent conditions where higher concentrations of suspended reef sediments are likely to have occurred.

### Settlement plates

Recruitment onto all settlement plates was limited. Percent cover ranged from 4–25%, with coverage lowest at tiles deployed adjacent to the Cu deposit (Site C: 6.7 ± 0.9% cover) compared with the two sets of reference plates positioned between DGT sites N2 and N3 (Site A - 10.8 ± 1.4%; Site B – 12.8 ± 2.6%; Fig. 2 and Table 2). The organisms growing on plates were classified into operational taxonomic units (OTUs) based on their appearance (morphology and colour). OTUs were used due to the difficulty of identifying invertebrate recruits to species level without bringing in additional taxonomic expertise or using genetic identification methods. Settlement plate data (counts and coverage) did not meet the assumptions of parametric analyses so non-parametric Kruskal-Wallis ANOVA were used to test for differences in settlement patterns between sites.

**Table 2:** Operational taxonomic units (OTUs) recorded on settlement plates.

Site	Total Cover (%)	Barnacle	Foraminifera	Tube worm	White branching bryozoan	White non-branching bryozoan	Pink branching bryozoan	Orange ascidian	Anemone	Hydroid (% cover)
A	10.75 ± 1.36	0	3.88 ± 1.92	0.25 ± 0.17	1 ± 0.33	1 ± 0.59	6.88 ± 1.44	0.13 ± 0.34	1.13 ± 0.76	0
B	12.75 ± 2.6	0	0.5 ± 0.5	1.63 ± 0.91	2.13 ± 0.83	1.25 ± 0.67	7.88 ± 1.6	1.88 ± 0.95	1.13 ± 0.48	0
C	6.67 ± 0.88	3.11 ± 1.27	16.11 ± 3.65	0.11 ± 0.11	0.44 ± 0.18	1.56 ± 0.78	0	0.44 ± 0.29	1.56 ± 0.63	1.11 ± 0.73
**K-W ANOVA (A + B vs. C)**										
	*	***	***	ns	ns	ns	***	ns	ns	*

Total plate coverage and mean (±one standard error) percentage cover and counts of OTUs are shown for each site. Data are the mean of nine replicate plates at a site. The significance of Kruskal-Wallis ANOVAs testing for differences between reference sites (A and B) and Cu impacted site C are indicated (ns = non-significant; *p < 0.1, ***p < 0.001).

When analyses were conducted to compare total plate coverage across the three sites (A vs. B vs. C) the observed differences were not significant (*p* > 0.1). However, when sites A and B were grouped to compare impact (site C) vs. reference sites it became apparent that invertebrate coverage was significantly lower on Cu impacted plates (*p* = 0.026). A comparison of OTU richness (number of different OTUs recorded on a plate) indicated that there was no difference in diversity between sites (Site A: 3.20 ± 0.40 (OTUs per plate ± s.e.), Site B: 3.60 ± 0.53 and Site C: 3.89 ± 0.39). While OTU richness did not vary between sites, there were clear differences in the communities recruiting between impact and reference sites. Barnacles and hydroids were only recorded on plates at the most Cu affected site (site C) while foraminifera were more abundant at site C than at reference sites (*p* < 0.001; Table 2). In contrast, pink branching bryozoans were present on plates at sites A and B but were not recorded on plates at site C. For other OTUs there were no apparent differences in abundance between treatments (Site C vs. Sites A and B).

## Discussion

### Distribution of Cu contamination at Otaiti

The multi-method Cu sampling and analysis strategy deployed here provides definitive proof of a discrete zone of water borne Cu contamination on Otaiti, Astrolabe Reef  ^6^ owing to the 7 to 12 tonnes of granulated Cu^2–8,10^ which still remain trapped beneath the wreck. While it is possible that some small proportion of Cu originated from the residual metal components of the *MV Rena*, we consider the Cu signal observed here primarily originated from the granulated Cu. Results of [_d_Cu_T_] measurements were much higher than [_d_Cu_T_] typically recorded in New Zealand coastal waters^32^ (generally <4 nM). The high [_d_Cu_T_] also equated to elevated [Cu’] and [Cu_DGT_], with the calculated values falling within the expected hierarchy for these techniques ([Cu_DGT_]» [_d_Cu_T_])^27^. Despite the very high [Cu_T_] encountered near the location with the highest sediment Cu concentrations, [Cu_T_] at Otaiti sites distal to the [Cu_T_] maximum were generally equal to, or less than the [Cu_T_] detected at the study control site of Okarapu Reef. This demonstrates that the plume of Cu in the Otaiti water column is localised around the wreck debris. Total dissolved Cu concentrations at all sites were higher than the ANZECC (2000) default trigger values for the protection of 99% of saltwater species, resulting in possible adverse effects for more sensitive species and early life stages. These ANZECC values, together with elevated [Cu_T_], [_d_Cu_T_], and [Cu_DGT_] and settlement plate data indicate that Cu contamination continues to affect the ecological composition of the Astrolabe Reef ecosystem even after a 5-year recovery from the grounding and breakup of the *Rena*.

### Cu speciation analysis

The data in the present study indicates the presence of only one organic ligand class with conditional stability constants of log*K* ≥ 11_._ These log*K* values fall within the range generally reported for L_2_^33,34^. The relatively narrow range of observed stability constants (log*K* = 11.6 ± 0.3) may indicate a relatively uniform composition of the ligands throughout the study area. Culture experiments with a number of species of planktonic mircoorganisms as well as with blue mussel embryos have shown that these organisms can produce Cu-binding ligands under Cu-stress^19,35–39^. For example, numerous species of marine cyanobacteria (e.g. *Synechococcus*)^19,35,36^, brown algae (e.g. *Fucus vesiculosus*)^37^ and coccolithophorids (e.g. *Emiliania huxleyi*)^38^ are known to produce Cu-chelators (log*K* > 11) in cultures, exuded as a feedback mechanism against the potential toxicity of Cu^2+^ ions^40,41^. However, it should be noted that ligands derived from terrestrial dissolved organic matter (DOM) (i.e., humic and fulvic acids)^26,42^, anthropogenic inputs (i.e., stormwater/wastewater)^43^, and antifouling paints^44^ are also potential sources of Cu-complexing ligands in the marine environment^40,45^. Further, resuspended sediments could add to the marine organic ligand pool, although the majority of studies have indicated that only weak ligands are derived from sediments (log*K* < 9)^46,47^. AdCSV does not inform ligand composition, beyond identifying the stability of the CuL complexes and it is thus not possible to constrain ligand sources. Statements on ligand provenance remain only assumptions based on a correlation (or the lack of a correlation) of [L] with other data in the surrounding water. However, that [L] always exceeded the [Cu_T_] strongly hints toward an active biological response by local biota to mitigate the toxic effects of the elevated Cu-levels around the *Rena* wreck. This inference is further supported by the fact that [Cu’] and [Cu_T_] were higher in the vicinity of the *Rena* Cu deposit, during one of the sampling dates, and the [L] followed that trend. Nevertheless, more research regarding the chemical nature of prevalent ligands and the possibility of ligand production by local benthic, planktonic, and microbial communities is needed to validate this conjecture.

The concentration of organic ligands can vary temporally as well as spatially based on a multitude of biotic and abiotic processes present in complex and heterogeneous natural waters, which influence the source as well as the concentration of prevalent organic ligands (e.g. DOM species)^48,49^. It is well known, that mixing of fresh- and marine waters, riverine discharge levels, hydrological conditions (i.e., currents and mixed layers), as well as meteorological conditions (i.e., precipitation, storm events, photochemical ligand destruction), anthropogenic input and biological processes (e.g. ligand production, microbiological oxidation) can spatially and temporally alter the ligand pool^49^ and associated Cu’ concentrations. Therefore, differences and fluctuations in [Cu’] and [L], as seen in the study area during the two sampling periods, are normal, especially in dynamic marine reef environments such as Otaiti.

[L] was always in excess of [_d_Cu_T_] in all samples, which reduced the [Cu’] in solution, but bioavailable [Cu’] measurements at Otaiti were often above 10^−11^ M (ranging from 2.3 to 81.9 pM), a threshold found to be toxic for some marine phytoplankton species^50,51^. Although [Cu’] recorded at Otaiti were two orders of magnitude lower than those calculated to be toxic to blue mussel embryos^39^, the settlement plates in the vicinity of the *Rena* Cu deposit showed lower benthic invertebrate recruitment. Additionally, different types of organisms were recruiting the settlement plates in high Cu’ areas. This finding illustrates that Cu’ sensitivity is species dependent with varying effects to a broad range of different marine organisms. Consequently, elevated [Cu’], both naturally and anthropogenically derived, can drive differences in organism recruitment or survival and thus alter community diversity, structure, and the functionality of affected ecosystems such as Otaiti^14,52^. Organisms unable to counteract persistent or intermittent high Cu’ exposures through ligand production, or other metabolic pathways, may struggle to compete with species that are more tolerant of Cu contamination. Furthermore, water samples in the present study were taken under calm conditions and thus represent a quiescent Cu baseline for the Otaiti environment. Sampling under calm conditions is likely to underestimate the [Cu_T_] encountered during storms (due to resuspension of Cu in sediments), which would likely increase [_d_Cu_T_] and [Cu’] in the water column thereby exceeding acute Cu-toxicity limits for various benthic biota, for at least short periods of time.

Future work could look at the chemical structures of organic Cu-binding ligands, the organisms and sources that produce them^41^ as well as the abiotic parameters influencing the ligand pool in the Otaiti aquatic environment to gain a deeper understanding of Cu-speciation and associated Cu-risks assessments in the vicinity of the *Rena* wreck.

### Settlement plate study

Although the spatial extent of the settlement plate deployment was limited compared to the deployment of DGTs, the results indicate an effect of Cu on the recruitment of benthic invertebrates. Not only was recruitment lower on plates positioned within the area of maximum [Cu_T_] and [Cu’] but there were differences in the types of organisms recruiting to Cu impacted plates. Some taxonomic groups (e.g. barnacles and foraminifera) were either restricted to, or more abundant, at the site of highest Cu contamination, leading to the interpretation that these organisms were more tolerant of Cu enriched water. Conversely, other OTUs (including bryozoans) were either restricted to reference sites, more abundant at reference sites, or showed no difference in abundance across the Cu contamination gradient. These results are consistent with previous studies identifying varied levels of Cu toxicity to a broad range of aquatic organisms^12,53–59^ including invertebrates, which can drive differences in benthic invertebrate recruitment and therefore community structure^14,52^.

Because of the limitations of the settlement plate study (limited replication and spatial distribution of sampling stations), the results should not be treated as the definitive assessment of the effects of sediment Cu contamination on the recruitment of benthic invertebrates to Otaiti. The experimental design was an economical one (in terms of time and resources), conducted in mid-winter, a time when recruitment was likely to be minimal and designed to give an indication of possible effects. Despite these caveats, the results do appear to confirm the *a priori* hypothesis that recruitment of invertebrates to hard substrates would be modified by waterborne Cu contamination arising from the *Rena*. The data indicate that both substrate coverage and community composition were affected. How these effects on recruitment will influence the ecology of sites adjacent to the zone of elevated Cu contamination is uncertain. Furthermore, this study only tested the effects of waterborne Cu contamination rather than the consequences of Cu within the recruitment substrate. In soft sediments where extremely high concentrations of Cu (up to 780 g kg^−1^)^7^ have been documented, it can probably be assumed that the ecological consequences are more severe. Water and sediment chemistry data indicate that any effects of Cu clove are localised to areas in the immediate vicinity of the Cu clove deposit. Nevertheless, the halo of [Cu’] at Otaiti could have significant localised effects in the long-term and could extend in a plume in various directions over the reef depending on conditions. While elevated [Cu] is restricted to a small proportion of the subtidal habitat available at Otaiti, the [Cu’] is present at or above the threshold for toxicity to some biota^50,51^ which may not be able to counteract Cu exposure through ligand production. Further experimentation and monitoring would be needed to better quantify the effects of Cu clove on recruitment and ecology, such as the potentially toxic effects of Cu on surface water phytoplankton communities. Without further research, the toxic or sub-lethal effects of Cu on reef biota and the long-term environmental consequences remain uncertain.

## Conclusion

In conclusion, the water sampling and analysis reported here, demonstrate that waterborne [Cu] are elevated at Otaiti in the vicinity of the Cu clove deposit, and are well above background [Cu] recorded at a comparable reference site. Impacted sites are likely to experience episodes of very high [Cu_T_] and [_d_Cu_T_] in the water column close to sediments, depending on the degree of sediment entrainment, and are likely to have consistently elevated [Cu’] (as indicated by AdCSV and DGT measurements which integrate the average water chemistry over several days). The results of our settlement plate study support the interpretation that localised Cu contamination of the water column is likely to modify the recruitment of benthic invertebrate species at this site. Thus, elevated [Cu_T_], [_d_Cu_T_], and [Cu’] in the vicinity of the *MV Rena* has the potential to modify the ecological composition of the Otaiti Reef ecosystem as it recovers from the shipwreck.

## Methods

### Water column Cu analysis

#### Sample collection

Water samples were collected from discrete locations along the 25 m contour of Otaiti using a team of SCUBA divers on the 16^th^ and 20^th^ of June 2016. The divers positioned the DGTs at the location of the water samples. DGTs remained *in-situ* for 4–5 days. Water samples were taken using a pre-acid-cleaned 50 mL polyethylene tube which was connected to a peristaltic pump housed inside a shipboard clean laboratory. Water was pumped for the equivalent of three tube volumes to flush any residual sample prior to sample collection inside a Class 100 laminar flow hood. Samples were pumped directly into pre-acid cleaned^60^ LDPE bottles (Low-density polyethylene; Nalgene Laboratory, Penfield, NY, United States) which were rinsed three times with the fresh sample and then filled with zero headspace. Samples reserved for _d_Cu_T_ and Cu-speciation analysis were filtered through a 0.2 μm cartridge-filter (AcroPak, Supor)). All samples were kept refrigerated at <5 °C prior to analysis with the exception of samples for AdCSV analysis, which were filled with a 5% headspace and kept frozen prior to analysis.

#### Sample treatment for total Cu analysis with the HR-SF-ICP-MS

Samples for total Cu were acidified with quartz distilled HCl to a concentration of 0.024 M, resulting in a pH of ~1.8. Samples were left for 1 month prior to further processing. Subsequently, a volume of 15 mL sample was pipetted into an acid cleaned FEP vial. An internal standard (indium and lutetium) at a concentration of 5 nM as well as H_2_O_2_ were added (final concentration 26 μM) prior to UV digestion^61^. Hydrogen peroxide was added to support the complete oxidation of organic matter in the sample under the influence of UV-light.

A seaFAST pico system was used in off-line mode to pre-concentrate the acidified seawater samples with a factor of 10. The eluate was subsequently transferred into an acid cleaned destination vial and was then ready for High Resolution Sector Field Inductively Coupled Plasma Mass Spectrometer (HR-SF-ICP-MS) analysis. A detailed description of the method and sample preparation procedure can be found in Biller and Bruland^62^, Lagerström, *et al*.^63^ and Middag, *et al*.^61^.

Samples were analysed using a Nu Attom HR-SF-ICP-MS utilizing wet plasma at a resolution setting of 4000. The system was calibrated using standard additions of a multi-element stock solution to seawater of low metal concentration. Quantitative recovery on the resin was verified by comparing the slope of the calibration line obtained from the standard additions to seawater to standard additions done directly to untreated eluent acid^61^. Recovery of the method was acceptable (>94%). The accuracy of the method was verified by the measurement of certified reference material (SLEW-3; National Research Council, Canada) and was found to be accurate within 4.6% of the reference value for Cu (reference value: 24.39 ± 1.89 nM Cu; measured value (*n* = 2): 25.52 ± 0.01 nM Cu).

#### DGT analysis

Diffusive gradients in thin films (DGT) provide a reliable direct, *in-situ* measurement of the concentration of the species of interest^64^. To determine the concentration of labile Cu in the water column at Otaiti, Chelex-100 DGT devices were prepared in-house after Zhang and Davison^64^. The precision and accuracy of the devices was confirmed before deployment and were found to be accurate within 5% for Cu with respect to [Cu_T_] in a 50 ppb standard solution (*n = 5*) deployment test. The assembled probes were immersed in 0.01M NaNO_3_ and transferred to the field in air-tight containers.

Four Chelex DGT probes were secured inside a robust plastic mesh container using cable ties. The container provided protection to the DGT probes from particles >5 mm diameter, whilst being completely permeable to seawater. Divers then transferred the container (attached to heavy weights) to the deployment location. Probes were co-located with two temperature loggers (UTBI-001, TidbiT V2 Temp Loggers) allowing the average DGT deployment temperature to be calculated at each respective depth.

After typically 4–5 days, the probes were retrieved, rinsed with deionised water (resistivity 18 MΩ) and kept in sealed, pre-cleaned individual plastic bags and were transferred to the lab at <5 °C in the dark. DGT probes were dismantled and Chelex-100 resins were eluted in 1 M ppt grade HNO_3_ and analysed using a Perkin Elmer (Waltham MA) quadrupole ICP-MS calibrated using certified NIST-traceable reference materials from Inorganic Ventures (Christiansburg, VA, USA) with an accuracy better than 1%^65^. Internal standards of known concentration were also analysed to determine instrumental drift during analysis. DGT blanks (HNO_3_ eluted Chelex resins) were below detection for Cu (<0.1 ppb). Reported [Cu_DGT_] were calculated using the standard DGT theory^6,29^ and Cu diffusion coefficients (determined from the average temperature over the deployment).

#### Voltammetric methods

Total dissolved Cu-concentrations and Cu-speciation were determined by voltammetric analysis using a Metrohm 663 VA stand connected to a PGSTAT10 (Eco Chemie) potentiostat interfaced with GPES v4.9 software. The three-electrode configuration of the system included a hanging mercury drop electrode (HMDE) as the working electrode, an Ag/AgCl^−^ 3M KCl reference electrode, and a platinum counter electrode. The system was operated in the differential pulse mode at room temperature. Instrumental settings were adopted from Sander, *et al*.^66^. For detailed information on sample preparation and operating conditions of the instrument see Sander, *et al*.^66^ and Powell^60^.

Acidified (q-HCl) aliqouts (4 mL) of each sample (pH: 1.7 ± 0.1) were transferred into acid cleaned Teflon vials. Samples were then UV-digested for at least 12 hours in order to remove natural Cu-complexing species in solution^60,67^. Once UV-digested each sample was pipetted into a trace metal clean and previously conditioned voltammetric cup. From this 0.01 M salicylaldoxime (Acros Organics; SA: 98%) was added to the sample to a final concentration of 25 µM and the sample was then left to equilibrate for 20 min. Salicylaldoxime was used as the added ligand, since its complexation parameters with Cu in seawater are well characterized^68^. From this, all samples were buffered to pH 8.1 ± 0.2 using 50 µL of 1M borate buffer (Arcos Organics; H_3_BO_3_: 99.99%) and 15 µL trace-metal grade NH_4_OH (Optima™, Fisher Chemical). The total dissolved Cu concentration in each sample was then determined by adsorptive cathodic stripping voltammetry (AdCSV)^67^. Prior to analysis, each sample aliquot was deaerated by purging nitrogen gas over the sample for at least 2 min^68–70^. The conditioning potential was set at −0.15 V for 15–30 s with a cathodic scan from −0.15 to −0.6 V. Every measurement was repeated three times^67^. After the first measurement a four-point standard addition procedure of a known Cu standard (Fisher Scientific) was used to subsequently determine the [_d_Cu_T_] of the sample^71^. The accuracy and precision of the voltammetric method was assessed by multiple measurements of a certified reference seawater sample^60,67^, i.e., SLEW-3. Accuracy and precision of the analytical technique was within an acceptable range of ± 3.5% SD for Cu (reference value: 24.39 ± 1.89 nM Cu; measured value (*n* = 2): 23.53 ± 1.45 nM Cu).

For the Cu-complexing ligand titrations, 12 aliquots (4 mL each) of each sample were separately transferred into pre-conditioned Teflon vials. Afterwards, 100 µL of 1M borate buffer was added to each sample to maintain the sample pH at 8.2 ± 0.2 during analysis. This was followed by increasing Cu-additions of an atomic absorption Cu-standard, calculated as a geometrically spaced series^72^, to each aliquot ranging from 0 to 550 nM^66^. A 20 min equilibration period was implemented to allow the Cu to bind with the natural organic ligands present in solution. Afterwards SA was added to the aliquots at a final concentration of 5 μM. Finally, all samples were left to equilibrate for a minimum of 12 hours, which was found to result in stable Cu–SA peaks^66^. Once equilibrated, all samples were analysed using the instrumental parameters as described for the total dissolved Cu-measurements. After completion of the titration, Cu-speciation parameters (i.e., [CuX_IN_], [Cu^2+^], [L], and log*K*) of each solution were obtained using the one-ligand and two-ligand complete complexation-fitting model within the ProMCC software^73,74^. Fitting of each titration data to a two-ligand system failed with the ProMCC software and thus samples were fitted to a one-ligand model, which resulted in reasonable at equilibrium speciation estimates of [L] and log*K* with derived parameters of [CuX_IN_] and [Cu^2+^]. Finally, the bioavailable [Cu’] was calculated as the sum of [CuX_IN_] and [Cu^2+^].

### Settlement plates

**Figure 7:**
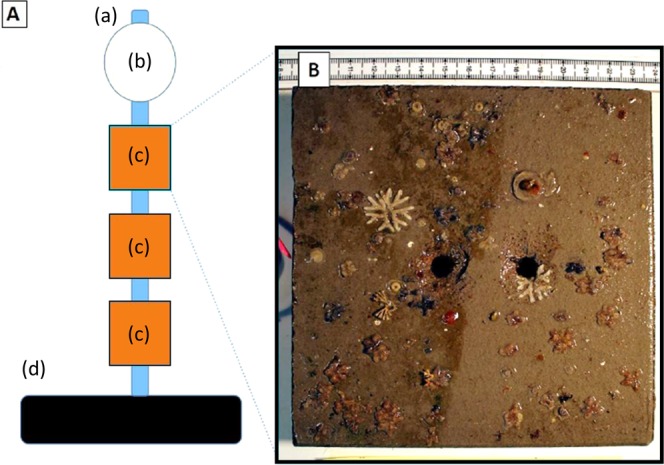
Panel A shows a schematic of settlement plate deployment unit including (a) polystyrene net float, (b) PVC pipe, (c) settlement plates and (d) 15 kg plastic coated weight. Three of these units were deployed at each settlement plate sampling station (Sites A, B and C). Panel B shows an example of a 15 × 15 cm terracotta settlement plate deployed at Otaiti to a depth of 25 m for 3 months.

The deployment of settlement plates was opportunistic and as such it was not possible to replicate the spatial arrangement of the DGT deployment. Instead, settlement plates were placed at the site of the Cu deposit (Site C) and at two sites (Sites A and B) between N2 and N3 (Fig. 2). Each settlement plate deployment unit consisted of three settlement plates (15 × 15 cm terracotta tiles) cable tied to a 1 m length of PVC pipe (Fig. 7). One end of the pipe was secured to a plastic coated 15 kg weight plate. The other end of the pipe was secured to a polystyrene net float. Three of these units (a total of nine settlement plates) were deployed at each site by SCUBA divers. After a period of 3 months, the plates were retrieved and photographed. Photographs were loaded into ImageJ (ImageJ, Ver. 1.49) and total plate coverage determined by summing the surface area of all parts of a plate covered by encrusting organisms. The OTUs used for comparing community composition between deployment sites were defined during this initial examination. OTUs definition was based on morphology (viewed under dissecting scope) and colour (Table 2). Once OTUs were defined, coverage (hydroids) of counts either individuals (barnacles and anemones) or colonies (bryozoans and ascidians) were determined for the entire plate. Kruskal-Wallis ANOVAs were performed in Statistica (Ver. 13) to test for differences in plate coverage between all sites (A vs. B vs. C) and between reference and impact sites (sites A and B vs. C). Subsequently, differences in counts and coverage of OTUs between reference and impact sites were assessed, again using Kruskal-Wallis ANOVAs.
